# 4-Chloro-*N*-(2,3-dimethyl­phen­yl)benzene­sulfonamide

**DOI:** 10.1107/S1600536811016321

**Published:** 2011-05-07

**Authors:** K. Shakuntala, Sabine Foro, B. Thimme Gowda

**Affiliations:** a Department of Chemistry, Mangalore University, Mangalagangotri 574 199, Mangalore, India; b Institute of Materials Science, Darmstadt University of Technology, Petersenstrasse 23, D-64287 Darmstadt, Germany

## Abstract

In the title compound, C_14_H_14_ClNO_2_S, the two aromatic rings are tilted relative to each other by 34.7 (1)°. In the crystal, the mol­ecules form zigzag chains along the *c* axis *via* inter­molecular N—H⋯O hydrogen bonds.

## Related literature

For hydrogen bonding modes of sulfonamides, see; Adsmond & Grant (2001 ▶). For our study of the effect of substituents on the structures of *N*-(ar­yl)-amides, see: Gowda *et al.* (2004 ▶); on the structures of *N*-(ar­yl)aryl­sulfonamides, see: Gowda *et al.* (2009 ▶); Shakuntala *et al.* (2011 ▶) and on the structures of *N*-(ar­yl)methane­sulfonamides, see: Gowda *et al.* (2007 ▶).
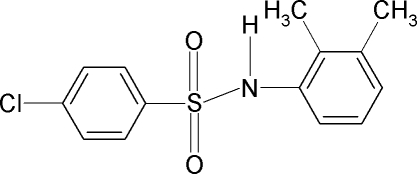


## Experimental

### 

#### Crystal data

C_14_H_14_ClNO_2_S
*M* *_r_* = 295.77
Monoclinic, 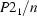

*a* = 4.9926 (6) Å
*b* = 22.296 (3) Å
*c* = 12.793 (2) Å
β = 90.11 (1)°
*V* = 1424.1 (3) Å^3^
*Z* = 4
Mo *K*α radiation
μ = 0.41 mm^−1^
*T* = 293 K
0.40 × 0.12 × 0.10 mm

#### Data collection

Oxford Diffraction Xcalibur diffractometer with a Sapphire CCD detector
Absorption correction: multi-scan (*CrysAlis RED*; Oxford Diffraction, 2009 ▶) *T*_min_ = 0.853, *T*_max_ = 0.960
5341 measured reflections
2669 independent reflections
1882 reflections with *I* > 2σ(*I*)
*R*_int_ = 0.021

#### Refinement

*R*[*F* ^2^ > 2σ(*F* ^2^)] = 0.055
*wR*(*F* ^2^) = 0.116
*S* = 1.07
2669 reflections
172 parameters
H-atom parameters constrained
Δρ_max_ = 0.47 e Å^−3^
Δρ_min_ = −0.40 e Å^−3^

### 

Data collection: *CrysAlis CCD* (Oxford Diffraction, 2009 ▶); cell refinement: *CrysAlis RED* (Oxford Diffraction, 2009 ▶); data reduction: *CrysAlis RED*; program(s) used to solve structure: *SHELXS97* (Sheldrick, 2008 ▶); program(s) used to refine structure: *SHELXL97* (Sheldrick, 2008 ▶); molecular graphics: *PLATON* (Spek, 2009 ▶); software used to prepare material for publication: *SHELXL97*.

## Supplementary Material

Crystal structure: contains datablocks I, global. DOI: 10.1107/S1600536811016321/bt5537sup1.cif

Structure factors: contains datablocks I. DOI: 10.1107/S1600536811016321/bt5537Isup2.hkl

Supplementary material file. DOI: 10.1107/S1600536811016321/bt5537Isup3.cml

Additional supplementary materials:  crystallographic information; 3D view; checkCIF report

## Figures and Tables

**Table 1:** Hydrogen-bond geometry (Å, °)

*D*—H⋯*A*	*D*—H	H⋯*A*	*D*⋯*A*	*D*—H⋯*A*
N1—H1*N*⋯O2^i^	0.86	2.46	2.893 (3)	112

Symmetry code: (i) 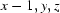.

**Fig. 1.**
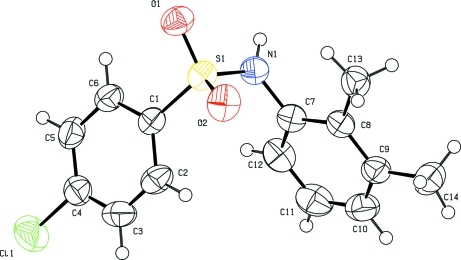
Molecular structure of (I), showing the atom labelling scheme. Displacement ellipsoids are drawn at the 50% probability level.

**Fig. 2.**
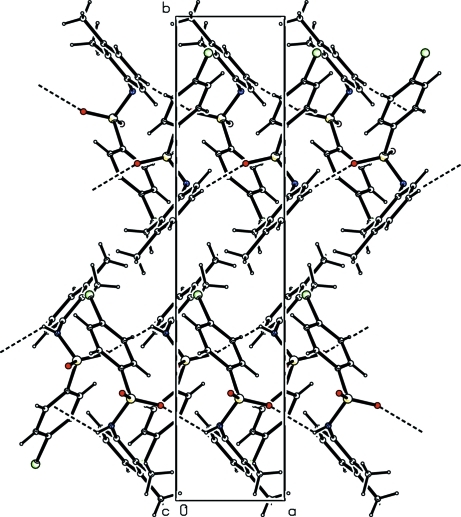
Molecular packing of (I) with hydrogen bonding shown as dashed lines.

## supplementary crystallographic information

### Comment

The sulfonamide moiety is a constituent of many biologically important compounds. The hydrogen bonding preferences of sulfonamides has been investigated (Adsmond & Grant, 2001). As a part of studying the substituent effects on the structures of this class of compounds (Gowda *et al.*, 2004, 2007, 2009; Shakuntala *et al.*, 2011), in the present work, the crystal structure of 4-chloro-*N*-(2,3-dimethylphenyl)-benzenesulfonamide, (I), has been determined (Fig. 1). In the title compound, the amino H atom is *trans* to one of the O atoms of the SO_2_ group. Furthermore, the N—H bond is *syn* to the *ortho*- and *meta*-methyl groups of the aromatic ring, in contrast to the *anti* conformation observed between the N—H bond, and the *ortho*- and *meta*-methyl groups in *N*-(2,3-dimethylphenyl)-benzenesulfonamide (II) (Gowda *et al.*, 2009). The molecule is twisted at the S atom with the C—SO_2_—NH—C torsion angle of -70.3 (3)°, compared to the values of 71.0 (2)° in (II), and -53.8 (3)° and -63.4 (3)° in the two independent molecules of 4-chloro-*N*-(phenyl)-benzenesulfonamide (III) (Shakuntala *et al.*, 2011).

The sulfonyl and the anilino benzene rings are tilted relative to each other by 34.7 (1)° in (I), compared to the values of 64.8 (1)° in (II), and 69.1 (1)° and 82.6 (1)° in the two independent molecules of (III).

The packing of molecules in the title compound *via* intermolecular N—H···O hydrogen bonds (Table 1) is shown in Fig. 2.

### Experimental

The solution of chlorobenzene (10 ml) in chloroform (40 ml) was treated dropwise with chlorosulfonic acid (25 ml) at 0 ° C. After the initial evolution of hydrogen chloride subsided, the reaction mixture was brought to room temperature and poured into crushed ice in a beaker. The chloroform layer was separated, washed with cold water and allowed to evaporate slowly. The residual 4-chlorobenzenesulfonylchloride was treated with 2,3-dimethylaniline in the stoichiometric ratio and boiled for ten minutes. The reaction mixture was then cooled to room temperature and added to ice cold water (100 ml). The resultant 4-chloro-*N*-(2,3-dimethylphenyl)-benzenesulfonamide was filtered under suction and washed thoroughly with cold water. It was then recrystallized to constant melting point from dilute ethanol. The compound was characterized by recording its infrared and NMR spectra.

Needle like colorless single crystals used in X-ray diffraction studies were grown in ethanolic solution by slow evaporation at room temperature.

### Refinement

The H atoms were positioned with idealized geometry using a riding model with N—H = 0.86 Å, the aromatic C—H = 0.93 Å, the methyl C—H = 0.96 Å, and were refined with isotropic displacement parameters set to 1.2 times of the *U*_eq_ of the parent atom.

### Crystal data

**Table:**

C_14_H_14_ClNO_2_S	*F*(000) = 616
*M**_r_* = 295.77	*D*_x_ = 1.380 Mg m^−^^3^
Monoclinic, *P*2_1_/*n*	Mo *K*α radiation, λ = 0.71073 Å
Hall symbol: -P 2yn	Cell parameters from 1658 reflections
*a* = 4.9926 (6) Å	θ = 3.2–27.9°
*b* = 22.296 (3) Å	µ = 0.41 mm^−^^1^
*c* = 12.793 (2) Å	*T* = 293 K
β = 90.11 (1)°	Needle, colourless
*V* = 1424.1 (3) Å^3^	0.40 × 0.12 × 0.10 mm
*Z* = 4	

### Data collection

**Table:**

Oxford Diffraction Xcalibur diffractometer with a Sapphire CCD detector	2669 independent reflections
Radiation source: fine-focus sealed tube	1882 reflections with *I* > 2σ(*I*)
graphite	*R*_int_ = 0.021
Rotation method data acquisition using ω and φ scans	θ_max_ = 25.7°, θ_min_ = 3.2°
Absorption correction: multi-scan (*CrysAlis RED*; Oxford Diffraction, 2009)	*h* = −4→6
*T*_min_ = 0.853, *T*_max_ = 0.960	*k* = −27→26
5341 measured reflections	*l* = −13→15

### Refinement

**Table:**

Refinement on *F*^2^	Primary atom site location: structure-invariant direct methods
Least-squares matrix: full	Secondary atom site location: difference Fourier map
*R*[*F*^2^ > 2σ(*F*^2^)] = 0.055	Hydrogen site location: inferred from neighbouring sites
*wR*(*F*^2^) = 0.116	H-atom parameters constrained
*S* = 1.07	*w* = 1/[σ^2^(*F*_o_^2^) + (0.0284*P*)^2^ + 1.6874*P*] where *P* = (*F*_o_^2^ + 2*F*_c_^2^)/3
2669 reflections	(Δ/σ)_max_ = 0.001
172 parameters	Δρ_max_ = 0.47 e Å^−^^3^
0 restraints	Δρ_min_ = −0.40 e Å^−^^3^

### Special details

**Table:**

Experimental. CrysAlis RED (Oxford Diffraction, 2009) Empirical absorption correction using spherical harmonics, implemented in SCALE3 ABSPACK scaling algorithm.
Geometry. All e.s.d.'s (except the e.s.d. in the dihedral angle between two l.s. planes) are estimated using the full covariance matrix. The cell e.s.d.'s are taken into account individually in the estimation of e.s.d.'s in distances, angles and torsion angles; correlations between e.s.d.'s in cell parameters are only used when they are defined by crystal symmetry. An approximate (isotropic) treatment of cell e.s.d.'s is used for estimating e.s.d.'s involving l.s. planes.
Refinement. Refinement of *F*^2^ against ALL reflections. The weighted *R*-factor *wR* and goodness of fit *S* are based on *F*^2^, conventional *R*-factors *R* are based on *F*, with *F* set to zero for negative *F*^2^. The threshold expression of *F*^2^ > σ(*F*^2^) is used only for calculating *R*-factors(gt) *etc*. and is not relevant to the choice of reflections for refinement. *R*-factors based on *F*^2^ are statistically about twice as large as those based on *F*, and *R*- factors based on ALL data will be even larger.

### Fractional atomic coordinates and isotropic or equivalent isotropic displacement parameters (Å^2^)

**Table:**

	*x*	*y*	*z*	*U*_iso_*/*U*_eq_	
C1	−0.0055 (6)	0.22651 (14)	0.3463 (2)	0.0406 (7)	
C2	0.1101 (6)	0.21646 (17)	0.2488 (3)	0.0536 (9)	
H2	0.2466	0.2413	0.2249	0.064*	
C3	0.0209 (7)	0.16946 (18)	0.1881 (3)	0.0573 (9)	
H3	0.0973	0.1623	0.1230	0.069*	
C4	−0.1806 (7)	0.13352 (15)	0.2240 (3)	0.0516 (9)	
C5	−0.2940 (7)	0.14244 (16)	0.3206 (3)	0.0544 (9)	
H5	−0.4285	0.1170	0.3443	0.065*	
C6	−0.2071 (6)	0.18932 (15)	0.3820 (3)	0.0490 (8)	
H6	−0.2837	0.1959	0.4472	0.059*	
C7	−0.0479 (6)	0.37146 (15)	0.2783 (2)	0.0426 (8)	
C8	0.1300 (6)	0.41952 (15)	0.2693 (2)	0.0425 (7)	
C9	0.1703 (6)	0.44421 (16)	0.1699 (3)	0.0487 (8)	
C10	0.0308 (8)	0.42138 (18)	0.0854 (3)	0.0612 (10)	
H10	0.0579	0.4379	0.0195	0.073*	
C11	−0.1469 (8)	0.37484 (19)	0.0965 (3)	0.0656 (11)	
H11	−0.2390	0.3603	0.0386	0.079*	
C12	−0.1889 (7)	0.34977 (17)	0.1933 (3)	0.0565 (9)	
H12	−0.3108	0.3186	0.2014	0.068*	
C13	0.2732 (7)	0.44507 (16)	0.3628 (3)	0.0551 (9)	
H13A	0.2268	0.4866	0.3706	0.066*	
H13B	0.4631	0.4414	0.3531	0.066*	
H13C	0.2211	0.4234	0.4244	0.066*	
C14	0.3601 (8)	0.49621 (18)	0.1552 (3)	0.0665 (11)	
H14A	0.5360	0.4849	0.1783	0.080*	
H14B	0.2988	0.5299	0.1953	0.080*	
H14C	0.3663	0.5069	0.0825	0.080*	
N1	−0.0959 (5)	0.34539 (12)	0.3801 (2)	0.0448 (7)	
H1N	−0.2211	0.3596	0.4190	0.054*	
O1	0.0057 (5)	0.27818 (11)	0.52703 (17)	0.0567 (6)	
O2	0.3577 (4)	0.30355 (11)	0.3984 (2)	0.0584 (7)	
Cl1	−0.2989 (3)	0.07532 (5)	0.14708 (8)	0.0827 (4)	
S1	0.08490 (15)	0.28949 (4)	0.42182 (7)	0.0439 (2)	

### Atomic displacement parameters (Å^2^)

**Table:**

	*U*^11^	*U*^22^	*U*^33^	*U*^12^	*U*^13^	*U*^23^
C1	0.0330 (16)	0.0457 (19)	0.0432 (18)	0.0034 (14)	0.0016 (13)	0.0061 (15)
C2	0.0417 (18)	0.066 (2)	0.053 (2)	−0.0004 (18)	0.0105 (16)	0.0081 (19)
C3	0.060 (2)	0.069 (3)	0.042 (2)	0.011 (2)	0.0078 (17)	−0.0032 (19)
C4	0.064 (2)	0.046 (2)	0.045 (2)	0.0038 (18)	−0.0099 (17)	0.0026 (16)
C5	0.062 (2)	0.047 (2)	0.054 (2)	−0.0121 (18)	0.0005 (17)	0.0084 (17)
C6	0.0497 (19)	0.052 (2)	0.0452 (19)	−0.0055 (16)	0.0075 (15)	0.0031 (16)
C7	0.0295 (16)	0.0493 (19)	0.0491 (19)	0.0046 (14)	0.0004 (14)	−0.0009 (16)
C8	0.0359 (17)	0.0448 (18)	0.0467 (19)	0.0048 (15)	0.0003 (14)	−0.0028 (15)
C9	0.0467 (19)	0.050 (2)	0.049 (2)	0.0086 (16)	0.0074 (16)	0.0002 (16)
C10	0.071 (2)	0.069 (3)	0.044 (2)	0.014 (2)	0.0033 (18)	0.0010 (19)
C11	0.064 (2)	0.076 (3)	0.057 (2)	0.008 (2)	−0.0156 (19)	−0.014 (2)
C12	0.044 (2)	0.058 (2)	0.067 (2)	−0.0031 (17)	−0.0097 (17)	−0.010 (2)
C13	0.060 (2)	0.049 (2)	0.057 (2)	−0.0072 (17)	−0.0039 (17)	−0.0010 (18)
C14	0.071 (3)	0.065 (3)	0.063 (2)	0.000 (2)	0.011 (2)	0.013 (2)
N1	0.0317 (13)	0.0481 (16)	0.0547 (17)	0.0026 (12)	0.0102 (12)	0.0003 (13)
O1	0.0572 (14)	0.0675 (16)	0.0453 (13)	−0.0098 (12)	−0.0013 (11)	0.0002 (12)
O2	0.0251 (11)	0.0680 (16)	0.0821 (18)	−0.0066 (11)	−0.0027 (11)	0.0055 (14)
Cl1	0.1197 (10)	0.0646 (7)	0.0637 (7)	−0.0041 (6)	−0.0174 (6)	−0.0123 (5)
S1	0.0286 (4)	0.0523 (5)	0.0510 (5)	−0.0050 (4)	0.0002 (3)	0.0027 (4)

### Geometric parameters (Å, °)

**Table:**

C1—C6	1.382 (4)	C9—C10	1.382 (5)
C1—C2	1.393 (4)	C9—C14	1.509 (5)
C1—S1	1.763 (3)	C10—C11	1.373 (5)
C2—C3	1.378 (5)	C10—H10	0.9300
C2—H2	0.9300	C11—C12	1.374 (5)
C3—C4	1.366 (5)	C11—H11	0.9300
C3—H3	0.9300	C12—H12	0.9300
C4—C5	1.375 (5)	C13—H13A	0.9600
C4—Cl1	1.732 (3)	C13—H13B	0.9600
C5—C6	1.377 (5)	C13—H13C	0.9600
C5—H5	0.9300	C14—H14A	0.9600
C6—H6	0.9300	C14—H14B	0.9600
C7—C12	1.382 (4)	C14—H14C	0.9600
C7—C8	1.397 (4)	N1—S1	1.628 (3)
C7—N1	1.447 (4)	N1—H1N	0.8600
C8—C9	1.401 (4)	O1—S1	1.426 (2)
C8—C13	1.505 (4)	O2—S1	1.430 (2)
			
C6—C1—C2	120.1 (3)	C9—C10—H10	119.3
C6—C1—S1	118.9 (2)	C10—C11—C12	120.1 (4)
C2—C1—S1	120.8 (3)	C10—C11—H11	120.0
C3—C2—C1	119.5 (3)	C12—C11—H11	120.0
C3—C2—H2	120.2	C11—C12—C7	119.2 (3)
C1—C2—H2	120.2	C11—C12—H12	120.4
C4—C3—C2	119.6 (3)	C7—C12—H12	120.4
C4—C3—H3	120.2	C8—C13—H13A	109.5
C2—C3—H3	120.2	C8—C13—H13B	109.5
C3—C4—C5	121.5 (3)	H13A—C13—H13B	109.5
C3—C4—Cl1	119.9 (3)	C8—C13—H13C	109.5
C5—C4—Cl1	118.6 (3)	H13A—C13—H13C	109.5
C4—C5—C6	119.5 (3)	H13B—C13—H13C	109.5
C4—C5—H5	120.2	C9—C14—H14A	109.5
C6—C5—H5	120.2	C9—C14—H14B	109.5
C5—C6—C1	119.7 (3)	H14A—C14—H14B	109.5
C5—C6—H6	120.1	C9—C14—H14C	109.5
C1—C6—H6	120.1	H14A—C14—H14C	109.5
C12—C7—C8	121.8 (3)	H14B—C14—H14C	109.5
C12—C7—N1	118.9 (3)	C7—N1—S1	120.7 (2)
C8—C7—N1	119.3 (3)	C7—N1—H1N	119.7
C7—C8—C9	118.0 (3)	S1—N1—H1N	119.7
C7—C8—C13	121.7 (3)	O1—S1—O2	120.18 (15)
C9—C8—C13	120.3 (3)	O1—S1—N1	106.86 (14)
C10—C9—C8	119.5 (3)	O2—S1—N1	106.92 (14)
C10—C9—C14	120.1 (3)	O1—S1—C1	107.77 (15)
C8—C9—C14	120.4 (3)	O2—S1—C1	107.62 (15)
C11—C10—C9	121.5 (4)	N1—S1—C1	106.81 (14)
C11—C10—H10	119.3		
			
C6—C1—C2—C3	0.4 (5)	C8—C9—C10—C11	−0.1 (5)
S1—C1—C2—C3	−175.1 (3)	C14—C9—C10—C11	−178.8 (3)
C1—C2—C3—C4	0.3 (5)	C9—C10—C11—C12	−0.2 (6)
C2—C3—C4—C5	−1.2 (5)	C10—C11—C12—C7	−0.8 (6)
C2—C3—C4—Cl1	178.7 (3)	C8—C7—C12—C11	2.1 (5)
C3—C4—C5—C6	1.3 (5)	N1—C7—C12—C11	179.2 (3)
Cl1—C4—C5—C6	−178.5 (3)	C12—C7—N1—S1	91.9 (3)
C4—C5—C6—C1	−0.6 (5)	C8—C7—N1—S1	−91.0 (3)
C2—C1—C6—C5	−0.3 (5)	C7—N1—S1—O1	174.6 (2)
S1—C1—C6—C5	175.3 (3)	C7—N1—S1—O2	44.7 (3)
C12—C7—C8—C9	−2.3 (5)	C7—N1—S1—C1	−70.3 (3)
N1—C7—C8—C9	−179.4 (3)	C6—C1—S1—O1	22.9 (3)
C12—C7—C8—C13	176.9 (3)	C2—C1—S1—O1	−161.6 (3)
N1—C7—C8—C13	−0.1 (4)	C6—C1—S1—O2	153.8 (3)
C7—C8—C9—C10	1.3 (5)	C2—C1—S1—O2	−30.6 (3)
C13—C8—C9—C10	−178.0 (3)	C6—C1—S1—N1	−91.7 (3)
C7—C8—C9—C14	180.0 (3)	C2—C1—S1—N1	83.9 (3)
C13—C8—C9—C14	0.7 (5)		

### Hydrogen-bond geometry (Å, °)

**Table:**

*D*—H···*A*	*D*—H	H···*A*	*D*···*A*	*D*—H···*A*
N1—H1N···O2^i^	0.86	2.46	2.893 (3)	112

Symmetry codes: (i) *x*−1, *y*, *z*.
